# Concomitant Enhancement of the Reorientational Dynamics of the BH_4_^–^ Anions and Mg^2+^ Ionic Conductivity in Mg(BH_4_)_2_·NH_3_ upon Ligand Incorporation

**DOI:** 10.1021/acs.jpcc.5c07031

**Published:** 2025-12-23

**Authors:** J. B. Grinderslev, M. B. Amdisen, S. Rosenqvist Larsen, B. A. Trump, M. Karlsson, W. Zhou, T. J. Udovic, Y. Cheng, T. Tominaga, T. R. Jensen, M. S. Andersson

**Affiliations:** † Interdisciplinary Nanoscience Center (iNANO) and Department of Chemistry, 1006 University of Århus, Langelandsgade 140, DK-8000 Århus C, Denmark; ‡ Department of Chemistry—Ångström Laboratory, 8097 Uppsala University, Box 538, SE-751 21 Uppsala, Sweden; § 96994 NIST Center for Neutron Research, National Institute of Standards and Technology, Gaithersburg, Maryland 20899-6102, United States; ∥ Department of Chemistry and Chemical Engineering, 11248 Chalmers University of Technology, SE-412 96 Göteborg, Sweden; ⊥ Neutron Scattering Division, 6146 Oak Ridge National Laboratory, Oak Ridge, Tennessee 37831, United States; # Research Center for Neutron Science and Technology, Comprehensive Research Organization for Science and Society (CROSS), Tokai, Ibaraki 319-1106, Japan

## Abstract

The addition of neutral ligand NH_3_ is known to increase the Mg^2+^ ionic conductivity in Mg­(BH_4_)_2_·NH_3_ as compared to the parent compound Mg­(BH_4_)_2_. Using inelastic neutron scattering, quasielastic neutron scattering, synchrotron X-ray powder diffraction, impedance spectroscopy, and density functional theory, the structure, the dynamics, and the Mg^2+^ ionic conductivity were investigated. The results show that the introduction of the NH_3_ ligand not only enhances the Mg^2+^ ionic conductivity but also significantly increases the reorientational mobility of the BH_4_^–^ anions. Thus, the results suggest that there may be a link between the two. Furthermore, the results show that Mg­(BH_4_)_2_·NH_3_ exhibits two coordination environments for the BH_4_^–^ anions, which act as either bridging or terminal anions, in contrast to Mg­(BH_4_)_2_, which only exhibits bridging anions. The different coordination environments in Mg­(BH_4_)_2_·NH_3_ lead to a clear difference in dynamics where the terminal anions have a much lower reorientational energy barrier (∼65 meV), as compared to the bridging anions (∼280 meV), and thus become dynamically active at much lower temperatures. The results show that the NH_3_ ligands also exhibit reorientational dynamics and that these are even faster than the dynamics of the BH_4_^–^ anions, with the NH_3_ ligands having a reorientational energy barrier of ∼10 meV. In addition to the reorientational dynamics, the NH_3_ ligands undergo quantum mechanical rotational tunneling below 50 K. In summary, this study provides a detailed characterization of both the structure and the dynamics of Mg­(BH_4_)_2_·NH_3_ and suggests that the rapidly reorienting terminal BH_4_^–^ anions may be behind the increased Mg^2+^ ionic conductivity upon ligand incorporation.

## Graphical abstract

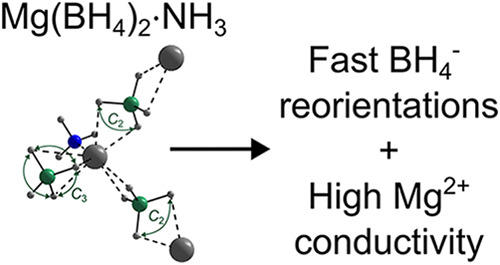

## 1 Introduction

Batteries have become a crucial technology in modern society, with applications spanning portable electronics, electric vehicles, and energy storage systems. However, significant advancements are needed to enhance battery performance in terms of higher energy density, improved safety, sustainability, and reduced costs to meet growing energy storage demands. Solid-state batteries present a promising solution to this challenge by enabling the use of metallic anodes and more efficient cell stacking. Moreover, solid-state electrolytes may improve battery safety due to their greater mechanical stability and lower flammability compared to the organic electrolytes in conventional lithium-ion batteries.^1–3^ Post-lithium-based technologies are receiving increased attention, due to both the limited abundance of lithium and the tendency for dendrite formation when using Li-metal. Magnesium-based batteries are a promising alternative, also offering a higher volumetric energy density of 3833 mAh cm^–3^ for Mg-metal as compared to Li-graphite (760 mAh cm^–3^) and Li-metal (2046 mAh cm^–3^).^4^ However, the high charge density of Mg^2+^ results in strong interactions with the anion lattice, often prohibiting high solid-state ionic conductivity.

There are only a few classes of solid materials that display high Mg^2+^ conductivity, limited to spinel-type chalcogenides, clay-like glassy electrolytes based on MgCl_2_–GaF_3_ composites, and a range of Mg­(BH_4_)_2_ derivatives.^5–7^ While the spinel-type chalcogenide MgSc_2_Se_4_ displays a high Mg^2+^ conductivity of around 10^–4^ S cm^–1^ at room temperature, it also suffers from a high electronic conductivity, hampering the applications as a solid electrolyte.^6,8,9^ A wide range of Mg­(BH_4_)_2_ derivatives have been investigated in recent years, revealing a multitude of new fast Mg^2+^ conductors using a variety of ligands, including NH_3_, CH_3_NH_2_, NH_2_CH_2_CH_2_NH_2_, (CH_3_)_2_CHNH_2_, NH_3_BH_3_, C_4_H_8_O, and (CH_3_OCH_2_CH_2_)_2_O, as the neutral ligand.^10–19^ Interestingly, eutectic mixtures can often be formed from composites of these compounds with different ligand contents, which significantly enhances the ionic conductivity and also results in soft clay-like electrolytes. These soft composites can be mechanically stabilized by metal oxide nanoparticles to obtain a solid nanocomposite electrolyte, which also prevents the recrystallization of the eutectic mixture and thereby preserves the highly conducting amorphous state to low temperatures.^10,14,20–22^ The application of these materials as solid electrolytes has also been demonstrated in all-solid-state batteries using a Mg-metal anode, a TiS_2_ cathode, and either Mg­(BH_4_)_2_·1.6NH_3_–MgO­(75 wt %) or Mg­(BH_4_)_2_·1.5C_4_H_8_O-MgO­(75 wt %) as the electrolyte.^10,23^

The mechanism behind the fast cationic conductivity in these materials is still not well understood and has mainly been investigated for the static average structure determined by powder X-ray diffraction as well as density functional theory (DFT) calculations to evaluate the energy landscape for a given conduction pathway.^11,24^ However, these methods fail to include the effects of the dynamics. Nuclear magnetic resonance (NMR) and quasielastic neutron scattering (QENS) are excellent probes for studying dynamics, in particular, for H-containing systems. This has been well-demonstrated, e.g., LiLa­(BH_4_)_3_X (X = Cl, Br, I), LiBH_4_–LiI solid-solutions, and LiBH_4_·NH_3_, where it was found that rapid BH_4_^–^ reorientations appear to be closely correlated to fast Li^+^ mobility through a concerted motion.^25–29^ Different Mg­(BH_4_)_2_ derivatives have also been investigated with QENS, e.g., amorphous and crystalline Mg­(BH_4_)_2_ (γ-polymorph), showing a higher ionic conductivity in the amorphous phase, which was attributed to a larger fraction of activated BH_4_^–^ rotations.^30^ A link between the BH_4_^–^ reorientations and Mg^2+^ conduction was also suggested for Mg­(BH_4_)_2_·0.5 (CH_3_OCH_2_CH_2_)_2_O, which contains two dynamically different BH_4_^–^ groups, assigned to a fast-reorienting terminal BH_4_^–^ group and a slow-reorienting bridging BH_4_^–^ group.^18^ A recent study on Mg­(BH_4_)_2_·CH_3_NH_2_ demonstrated a significantly higher reorientational frequency of BH_4_^–^ as compared to pristine Mg­(BH_4_)_2_, which correlates well with a high conductivity of σ­(Mg^2+^) = 2.1 × 10^–5^ S cm^–1^ at room temperature for the former, as compared to σ­(Mg^2+^) = 5.3 × 10^–14^ S cm^–1^ at 313 K for the latter.^30,31^ Moreover, it was found that the CH_3_ also underwent rapid rotations, while the NH_2_ was more restrained, likely due to the coordination to Mg^2+^.^31^

Here, we investigate the polymorphism, ionic conductivity, and dynamics of monoammine magnesium borohydride, Mg­(BH_4_)_2_·NH_3_, using inelastic neutron scattering (INS), QENS, synchrotron X-ray powder diffraction (SR-PXD), electrochemical impedance spectroscopy (EIS), and DFT.

## 2 Experimental Section

### 2.1 X-ray Powder Diffraction

In situ time-resolved temperature-varied SR-PXD data were collected at beamline BM01 at the European Synchrotron Radiation Facility (ESRF), Grenoble, France, on a DECTRIS PILATUS2M area detector with λ = 0.7980 Å. The sample was packed in a 0.5 mm borosilicate capillary, sealed in an argon atmosphere, and cooled using an Oxford cryostream.

High-resolution SR-PXD data were used for structural solution and refinements of the new low-temperature polymorph of Mg­(BH_4_)_2_·NH_3_, using the software FOX and Fullprof, respectively.^32,33^ The BH_4_^–^ and NH_3_ groups were treated as rigid bodies in FOX and by using bond-length restraints in Fullprof. The background was described by linear interpolation between selected points, while Pseudo-Voigt profile functions were used to fit the diffraction peaks. The experimentally determined structure was subsequently reoptimized by DFT to determine the orientations of the BH_4_^–^ anions and the NH_3_ ligands. The DFT optimization only resulted in minor changes to the atomic positions.

### 2.2 Neutron Spectroscopy

The neutron experiments were carried out using the Filter Analyzer Neutron Spectrometer (FANS)^34^ and the High Flux Backscattering Spectrometer (HFBS)^35^ instruments at the NIST Center for Neutron Research (NCNR), as well as using the Biomolecular Dynamics Spectrometer (BL02 DNA)^36^ at the Japan Proton Accelerator Research Complex (J-PARC). The reorientational dynamics were investigated using HFBS and DNA, while the vibrational dynamics were investigated using FANS. For HFBS, the instrument was configured to probe an energy window of ± 15 μeV with a resolution of ∼0.8 μeV and a Q-range of ∼0.4 to 1.65 Å^–1^. For DNA, the QENS spectra were measured using either the Si111 analyzer with an energy window of −40 to 50 μeV, a resolution of ∼3.6 μeV, and a Q-range of ∼0.2 to 1.8 Å^–1^, or the Si311 analyzer with an energy window of −150 to 200 μeV, a resolution of ∼19 μeV, and a Q-range of ∼1.75 to 3.7 Å^–1^. FANS covers an energy range of ∼5–250 meV with an energy resolution of ∼3%. For HFBS and FANS, ∼0.2 g of Mg­(^11^BH_4_)_2_·NH_3_ was distributed into an Al-foil sachet that was rolled up and inserted into an annular Al sample cell, which was sealed using an In O-ring. The preparation steps were carried out in a dry He glovebox. For the experiments using DNA, the same sample preparation steps were carried out, but using ∼0.1 g of Mg­(^11^BH_4_)_2_·NH_3_ powder and a steel O-ring. For DNA, QENS spectra were collected using the Si111 analyzer at 10 and 20 K, and then at every 15 K up to 305 K. The spectra collected at 10, 20, 95, 110, 125, 140, 155, 230, 245, and 260 K were collected for a longer period of time to allow for detailed fitting, while the spectra collected at the remaining temperatures were short and were used to determine the Elastic and Inelastic Fixed Window Scans (EFWS and IFWS) as described in more detail below. Detailed QENS spectra were also collected using the Si311 analyzer at 10, 95, 110, 125, 140, 155, 230, and 260 K. For HFBS, detailed QENS spectra were collected at 5, 225, and 250 K. For both HFBS and DNA, a vanadium sample was used to measure the instrumental resolution functions. Due to the large incoherent neutron scattering cross section of H (∼80 barns) as compared to the other elements (Mg ∼0.08, ^11^B ∼0.21, and N ∼0.5 barns), the signal was completely dominated by the scattering from H. The obtained spectra from a QENS experiment can be described by the scattering function

1 $$S\left(Q,\omega \right)=R\left(Q,\omega \right)⊗\left[\delta \left(\omega \right)A_{E}\left(Q\right)+\sum L_{i}\left(\omega \right)A_{\mathrm{QE},i}\left(Q\right)\right]+\mathrm{Bkg}\left(Q\right)$$

where *E* = ℏω is the neutron energy transfer, ℏ is the Planck constant/(2π), ω is the angular frequency, δ is a delta-function, *L*_*i*_’s are Lorentzian functions used to describe the quasielastic scattering, *A*_E_ and *A*_QE,*i*_ are the areas corresponding to the respective delta and Lorentzian functions, respectively, *R*(*Q*, ω) is the instrument resolution function, and Bkg­(*Q*) is a linear background.

### 2.3 Computational Modeling

The reorientational energy barriers for the NH_3_ ligand and the BH_4_^–^ anions were estimated from DFT calculations using the Vienna Ab initio Simulation Package (VASP)^37^ and the climbing image nudged elastic band (cNEB) method^38^ for the low-temperature structure (*P*2_1_2_1_2_1_). The DFT calculations used the projector augmented wave (PAW) method^39,40^ to describe the effects of core electrons. The optB86b-vdW functional^41,42^ with dispersion corrections was applied. The energy cutoff was 800 eV for the plane-wave basis of the valence electrons. The total energy tolerance was 10^–8^ eV for electronic energy minimization and 10^–7^ eV for structure optimization. The maximum interatomic force in the equilibrium configuration after relaxation was below 0.001 eV/Å. The cNEB calculation used 7 images, and the spring constant was −5 eV/Å^2^ (the negative sign means nudging is turned on).

For comparison with the experimental INS data, the phonon density of state (PDOS) was calculated for the low-temperature structure using the Quantum ESPRESSO package^43^ and the finite differences method and was appropriately weighted to account for the total neutron scattering cross sections of the different elements.

### 2.4 Electrochemical Impedance Spectroscopy

EIS data were collected by using a BioLogic MTZ-35 impedance analyzer equipped with a high-temperature sample holder. The sample was pressed into a 5 mm diameter pellet with a thickness of 0.60 mm under a pressure of 1 GPa for 2 min. EIS data were measured in a frequency range from 1.1 MHz to 1.1 Hz with an amplitude of 10 mV in the temperature range of 263 to 333 K.

## 3 Results and Discussion

### 3.1 Structural Characterization

In situ SR-PXD data of Mg­(BH_4_)_2_·NH_3_ was measured during cooling, revealing a second-order phase transition in the temperature range 280–260 K, see Figure 1. The differences in the diffraction pattern are subtle and are mainly associated with a change in the peak positions, but the occurrence of low-intensity peaks at 2θ = 14.8, 17.2, and 21.6° reveals that the symmetry is lowered from the room-temperature polymorph (*Pnma*) to a new low-temperature polymorph (*P*2_1_2_1_2_1_). Upon further cooling to 150 K, no significant changes are observed. The Bragg reflections from SR-PXD data of Mg­(BH_4_)_2_·NH_3_ could be indexed in the orthorhombic space group *P*2_1_2_1_2_1_ with unit cell parameters *a* = 11.2895(3), *b* = 7.4833(2), *c* = 6.7276(1) Å and *V* = 568.36(2) Å^3^ at 150 K. Structural solution revealed that the structure retains the same structural prototype as the room-temperature (RT) polymorph (*Pnma*) with zigzag chains along the *b*-axis consisting of tetrahedral [Mg­(NH_3_)­(BH_4_)_3_] complexes, which are connected via two bridging [BH_4_]^−^ units, see Figure 2. The terminal [BH_4_]^−^ group coordinates via face-sharing (κ^3^), the bridging groups via edge-sharing (κ^2^), while NH_3_ coordinates via the lone-pair on N, providing a coordination number of 8 for Mg^2+^. The Mg–B distances are 2.28 Å for the terminal [BH_4_]^−^ group and 2.44–2.47 Å for the bridging group, while the Mg–N bond distance is 2.08 Å, in good agreement with the RT-polymorph.^11^ The bridging [BH_4_]^−^ units in the RT-polymorph are described as a cubic arrangement of H around the B-site with 50% occupancy, suggesting that two different orientations of edge-coordinating [BH_4_]^−^ are present, while the low-temperature polymorph can be described with a single well-defined orientation.

**Figure 1:**
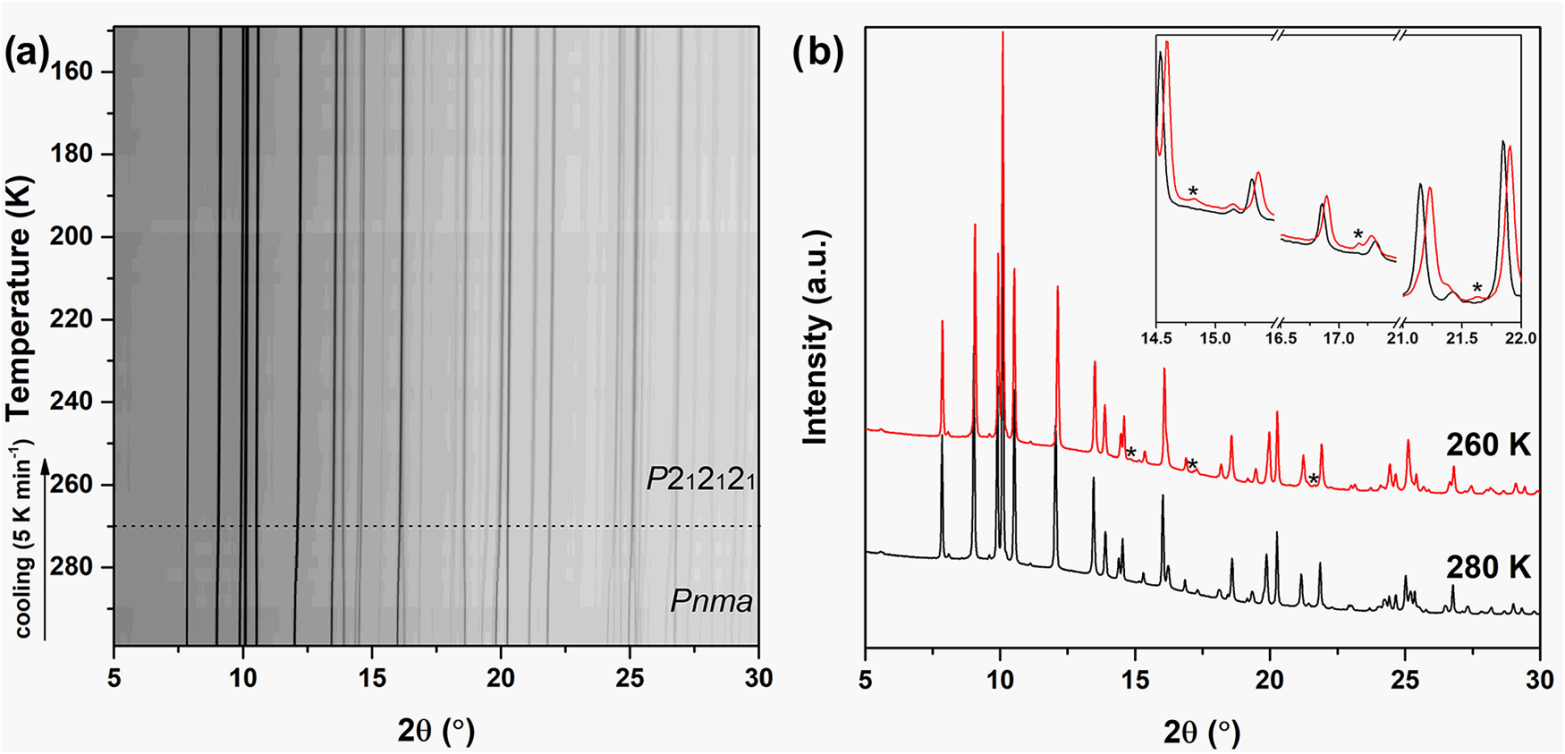
(a) In situ SR-PXD data of Mg­(BH_4_)_2_·NH_3_ during cooling from 297 to 150 K (Δ*T* = 5 K/min) and λ = 0.7980 Å. (b) SR-PXD data at 280 and 260 K. The new peaks from the *P*2_1_2_1_2_1_ polymorph are indicated with ‘*’ in the inset.

**Figure 2:**
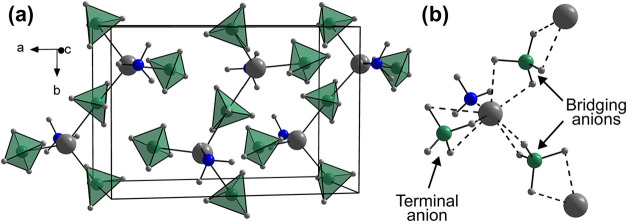
Crystal structure (a) and local Mg^2+^ coordination (b) of the low-temperature polymorph of Mg­(BH_4_)_2_·NH_3_ (*P*2_1_2_1_2_1_). Color scheme: Mg^2+^ (gray), N (blue), B (green), H (light gray), and [BH_4_]^−^ (green tetrahedra).

### 3.2 Inelastic Neutron Scattering

The INS spectra for Mg­(BH_4_)_2_·NH_3_ at 5 K, as well as DFT calculated spectra for Mg­(BH_4_)_2_·NH_3_, are presented in Figure 3. The good agreement between the measured and calculated spectra further reinforces the validity of the low-temperature structure shown in Figure 2, upon which the calculations are based. The spectrum can be divided into several parts. The low-energy regime (∼5–35 meV) is dominated by collective librations, which involve the movement of large Mg­(BH_4_)_2_·NH_3_ units. Stretches of the Mg^2+^—NH_3_ and Mg^2+^—BH_4_^–^ bonds occur in the energy range of ∼45–60 meV. At about 65 and 75 meV, there are two intense peaks; the peak at 65 meV is related to BH_4_^–^ librations, while the peak at 75 meV is related to NH_3_ librations. For the BH_4_^–^ librational, a peak at 65 meV and a shoulder at ∼64 meV can be observed, which corresponds to a mixture of bridging and terminal BH_4_^–^ anion librations, while the main peak at 66 meV is dominated by BH_4_^–^ librations from the bridging anions. BH_4_^–^ bending modes can be observed between 125 and 145 meV, while a mixture of BH_4_^–^ and NH_3_ bending modes dominates the spectra between 150 and 200 meV. No BH_4_^–^ or NH_3_ stretches can be observed in the studied energy range, and the first stretches are predicted to occur above 275 and 400 meV for BH_4_^–^ and NH_3_, respectively. The INS spectrum for Mg­(BH_4_)_2_·NH_3_ has many similarities to the INS spectra of Y­(BH_4_)_3_·*x*NH_3_ (*x* = 0, 3, or 7),^44^ which exhibit librational modes as well as stretches of the Y^3+^—BH_4_^–^ and Y^3+^—NH_3_ bonds below 130 meV. The Y­(BH_4_)_3_·*x*NH_3_ (*x* = 0, 3, or 7) spectra also exhibit BH_4_^–^ bending modes above ∼130 meV and a mixture of BH_4_^–^and NH_3_ bending modes above ∼150 meV. The measured spectrum also has a large overlap with the spectra for Mg­(BH_4_)_2_ (α, β, and γ),^45,46^ which exhibit librational BH_4_^–^ modes between 20 and 80 meV with an intense librational mode at about 65 meV, as well as BH_4_^–^ bending modes at ∼120–190 meV.

**Figure 3:**
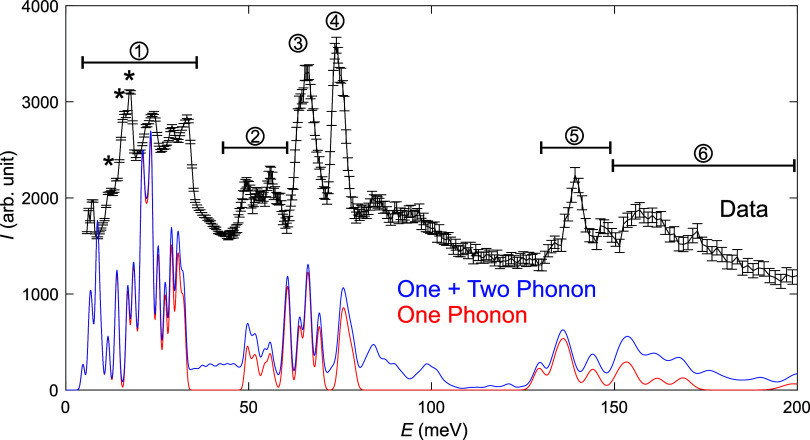
INS spectra of Mg­(BH_4_)_2_·NH_3_. (1) Collective librations involving Mg^2+^, NH_3_, and BH_4_^–^. (2) Stretches of the Mg^2+^—NH_3_ and Mg^2+^—BH_4_^–^ bonds. (3) BH_4_^–^ librations. (4) NH_3_ librations. (5) BH_4_^–^ bending modes. (6) Mixture of BH_4_^–^ and NH_3_ bending modes.

### 3.3 Quasielastic Neutron Scattering

#### 3.3.1 EFWS and IFWS

To investigate the development of the dynamics in Mg­(BH_4_)_2_·NH_3_, QENS spectra were measured at select temperatures between 10 and 305 K by using DNA and the Si111 analyzer. From these QENS spectra, the Elastic and Inelastic Fixed Window Scan intensities *I*_EFWS_ and *I*_IFWS_ were extracted by integrating select energy regions of the spectra, ±3.5 μeV (EFWS) and 14 to 21 μeV (IFWS). In an EFWS experiment, the intensity of elastic scattering is probed. Upon heating, dynamics on the time scale of the instrument may occur, which leads to a decrease in the elastic intensity as quasielastic broadening emerges from the elastic peak. Upon further heating, the dynamics may become too fast for the instrument to detect, which leads to a flattening out of the *I*_EFWS_ curve. For an IFWS experiment, the concept is similar. However, since the window of integration is placed with an offset from the elastic peak, the *I*_IFWS_ will start to increase as the quasielastic broadening enters the window of integration. As the dynamics becomes more rapid, it will eventually result in a decrease in the *I*_IFWS_ as the intensity gets distributed over an increasingly wider energy range. A more detailed description of EFWS and IFWS is given in refs 47,48 . For the EFWS of Mg­(BH_4_)_2_·NH_3_ presented in Figure 4(a), there are at least two regions that exhibit more pronounced drops in *I*_EFWS_. The first drop starts at ∼40 K and starts to flatten out around 170 K, and the second drop starts to occur around 200 K and potentially starts to flatten out close to 300 K. In addition, there is a weak indication of a small change in the slope around 80 K, which could indicate that the drop in intensity between 40 and 150 K is due to two different dynamical motions entering the time scales of the instrument. From the IFWS presented in Figure 4(b), it becomes clear that there are at least three regions of active dynamics as indicated by the three peaks at 65, 170, and 260 K. Based on previous studies of Y­(BH_4_)_3_·*x*NH_3_ (*x* = 0, 3 or 7),^49^ it is likely that the lowest-temperature dynamics is related to the NH_3_ ligand, while the higher-temperature dynamics is related to the bridging and terminal BH_4_^–^ anions. Due to the difference in local environment, the anions can be expected to have different dynamics; see Figure 2.

**Figure 4:**
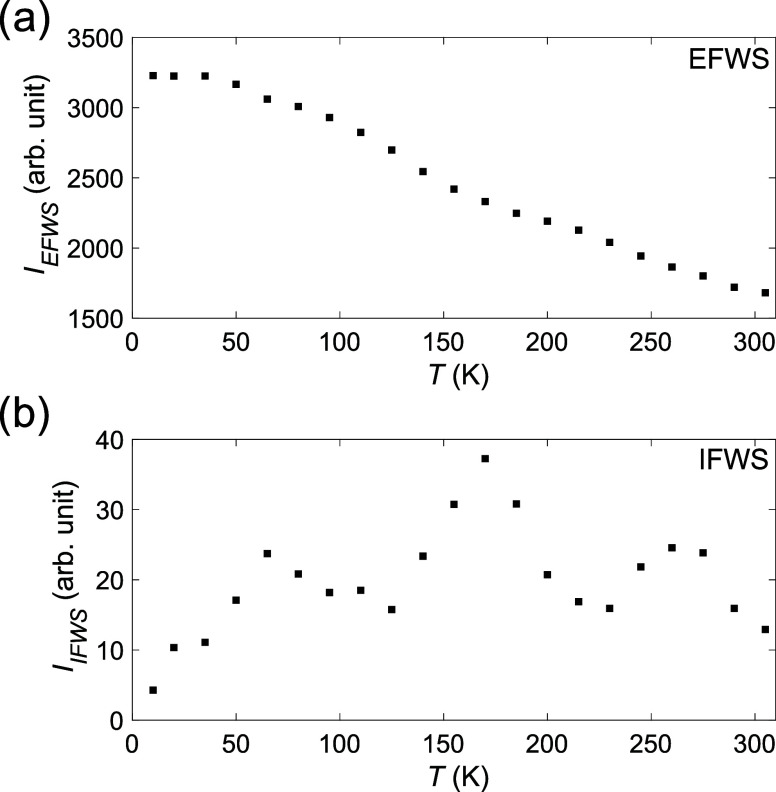
(a) EFWS and (b) IFWS for Mg­(BH_4_)_2_·NH_3_. The energy windows of integration were (a) ± 3.5 μeV and (b) 14 to 21 μeV.

#### 3.3.2 General Overview and Fitting of the QENS Spectra

In Figure 5, which presents QENS spectra at select temperatures between 10 and 305 K, two things can be clearly seen. First, the spectra at 10 and 50 K exhibit a satellite feature on each side of the elastic peak (indicated by black arrows in Figure 5). These features are often indicative of quantum mechanical rotational tunneling.^47,50–52^ The satellite features are discussed in more detail in the section about quantum mechanical rotational tunneling. Second, all spectra above 50 K exhibit clear quasielastic broadening, which is also reflected in a decrease of the elastic peak with increasing temperature. Thus, the QENS spectra indicate that there is active dynamics at all temperatures from 10 to 305 K.

**Figure 5:**
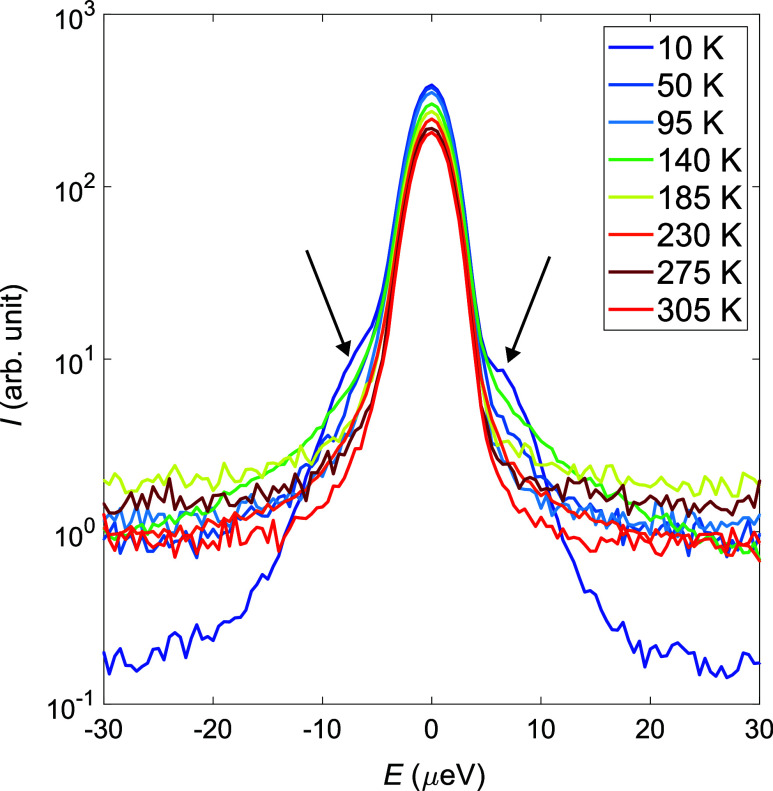
QENS spectra at selected temperatures over the temperature interval 10 to 305 K collected using DNA and the Si111 analyzer. The spectra correspond to a single Q-bin ranging from ∼0.2 to ∼1.8 Å^–1^. The black arrows indicate the satellite features that appear at low temperatures.

The detailed QENS spectra measured at 225 and 250 K from HFBS; 95, 110, 125, 140, 155, 230, and 260 K from DNA (Si111); and 95, 110, 125, 140, 155, 230, and 260 K from DNA (Si311) were fitted with eq 1 using PAN, which is part of the DAVE distribution;^53^ see Figure 6(a) and (b). At 95 K, the Si311 spectra already contain a wide component, suggesting that the dynamics that enters the experimental time window at ∼50 K reflects very rapid motions already at 95 K. This component gradually develops also in the Si311 spectra at 110, 125, 140, and 155 K; however, at these temperatures a second Lorentzian component is also needed to describe the spectra. This much narrower component is also seen in the high-resolution Si111 data at 110, 125, 140, and 155 K and suggests that a dynamical motion enters the experimental time window at 110 K. The data from HFBS and DNA Si111 at 225, 230, 245, 250, and 260 K can be described by a single narrow quasielastic component; however, this component is about 10 and 100 times more narrow than the two components detected at 155 K, suggesting that a third dynamical motion enters the time window on the instrument. The DNA Si311 spectra at 230 and 260 K can be accurately described using two quasielastic components, a narrow component which is the same as seen in the HFBS and DNA Si111 spectra at 225, 230, 245, 250, and 260 K and a much wider component that likely corresponds to the two components detected at 155 K, which have become much broader with the increase in temperature and are no longer possible to distinguish from one another. These observations are in good agreement with the findings from the EFWS and IFWS analyses, which suggest that a first dynamical motion becomes available above ∼50 K, followed by a second dynamical motion at about ∼100 K and finally a third dynamical motion above ∼200 K. As discussed in connection with the EFWS and IFWS results, the fast dynamics present at 95 K is likely from the NH_3_ ligand, while the two other dynamical motions present at higher temperatures are likely from the BH_4_^–^ anions with different local environments (terminal and bridging); see Figure 3. The full width at half-maximum (FWHM) of all Lorentzian components is roughly Q-independent, suggesting that the observed dynamics are likely due to local reorientations; see Figure 6(c,d). This is in good agreement with previous QENS and NMR studies on borohydrides.^49,54–57^

**Figure 6:**
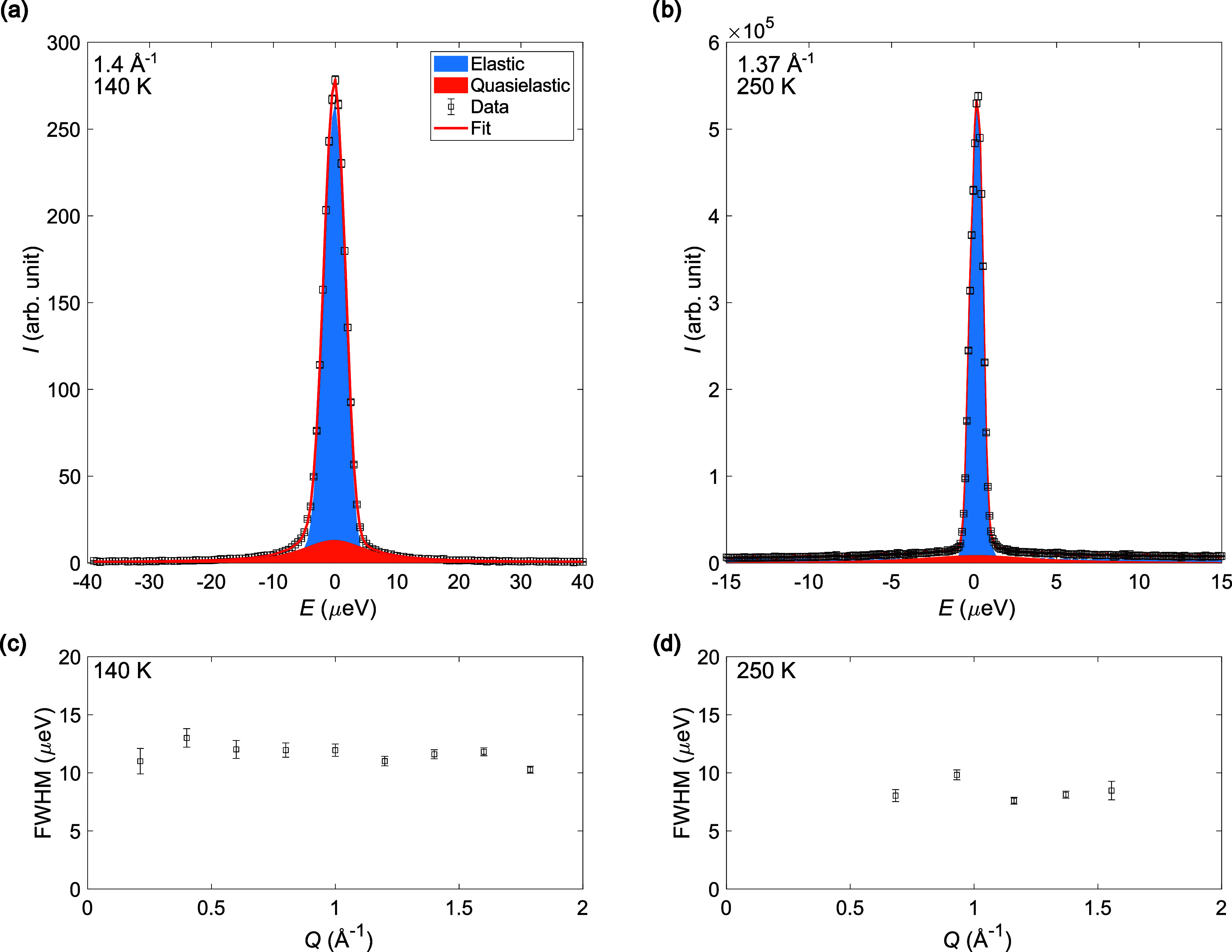
Fits of the QENS spectra at (a) 140 K (DNA Si111) and (b) 250 K (HFBS) for a selected *Q*-value with the individual elastic and quasielastic components as well as the total fit to the data. FWHM of the quasielastic component as a function of *Q* at (c) 140 K (DNA Si111) and (d) 250 K (HFBS), i.e., the same spectra as shown for a single *Q*-value in (a, b).

#### 3.3.3 Reorientational Motions of the NH_3_ Ligands and BH_4_^–^ Anions

Using the determined elastic and quasielastic contributions for the fits to eq 1, the Elastic Incoherent Structure Factor (EISF) was estimated using

2 $$\mathrm{EISF}=\frac{A_{E}\left(Q\right)}{A_{E}\left(Q\right)+\sum A_{\mathrm{QE},i}\left(Q\right)}$$

The experimental EISF at 95, 140, and 230 K, together with EISF models for different reorientational motions of the NH_3_ ligands and BH_4_^–^ anions in Mg­(BH_4_)_2_·NH_3_, are presented in Figure 7. At 95 K, where only one motion is expected to be active based on the EFWS, IFWS, and fitting of the QENS spectra, the experimental EISF agrees well with a model that considers 3-fold rotation around the *C*_3_ axis of the NH_3_ ligands with the BH_4_^–^ anions being dynamically frozen. The width of the single component at 95 K matches the resolution and the energy window of both Si111 and Si311. Thus, the experimental EISF can be extracted over a wide Q-range for this temperature. However, at 140 and 230 K, at least one component is too wide, as compared to the instrumental energy window of Si111, to be accurately determined, and thus, no experimental EISF can be determined using Si111 for these temperatures. At 140 K, where two motions are expected to be active and the best agreement is found between the data and an EISF model which takes into account 3-fold rotations around the *C*_3_ axis of all of the NH_3_ ligands and 2- or 3-fold rotations around the *C*_2_ or *C*_3_ axes of 50% of the BH_4_^–^ anions, with the remaining BH_4_^–^ anions dynamically frozen. As shown in the Supporting Information (SI), the EISF for 2-fold rotations around the *C*_2_ axis is identical to 3-fold rotations around the *C*_3_ axis for tetrahedral anions such as BH_4_^–^, and it is thus not possible to distinguish 2- and 3-fold reorientations of the BH_4_^–^ anion from the EISF alone. At 230 K, where a third dynamical motion is expected to have entered the time window of the instrument, the best agreement is found between the experimental EISF and an EISF model that takes into account 3-fold rotations of all of the NH_3_ ligands and 2-fold and/or 3-fold rotations of all of the BH_4_^–^ anions. Comparing the findings of the EISF with the determined structure presented in Figure 2, it can be noted that there are two types of BH_4_^–^ anions: terminal (50%) and bridging (50%). As seen in Figure 2(b), the bridging BH_4_^–^ anion will form a linear Mg-BH_4_–Mg axis, around which the anion can rotate without breaking the bonds to either of the two Mg^2+^ cations. In a similar manner, the terminal BH_4_^–^ anions orient three of their hydrogens toward the Mg^2+^ cation and can thus rotate freely around its *C*_3_ axis without breaking any bonds. Furthermore, the NH_3_ ligand is orientated away from the Mg^2+^ cation so that its three hydrogen atoms can freely rotate around its *C*_3_ axis; see Figure 2. The findings from the EISFs are thus in excellent agreement with both the determined crystal structure for Mg­(BH_4_)_2_·NH_3_ and the EFWS and IFWS results. Moreover, they are also in good agreement with previous studies of other borohydride ligand systems, such as Mg­(BH_4_)_2_·CH_3_NH_2_ and Y­(BH_4_)_3_·*x*NH_3_ (*x* = 0, 3, or 7).^31,49,57^

**Figure 7:**
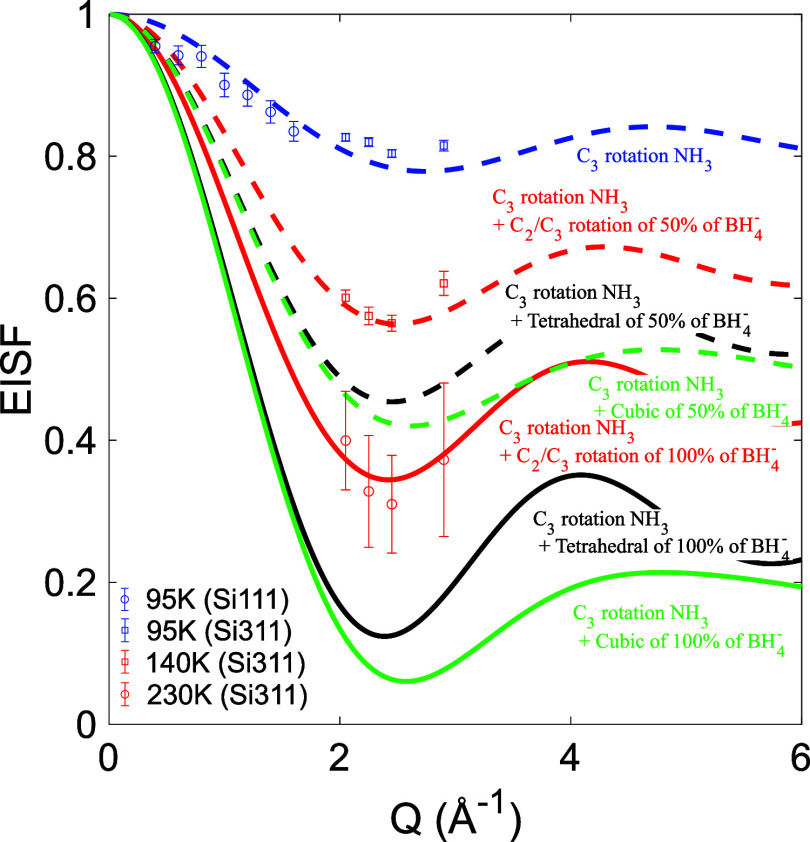
EISF extracted from the QENS spectra at 95, 140, and 230 K (DNA Si311) with corresponding EISF models. Details about the different EISF models are given in the SI.

#### 3.3.4 Reorientational Energy Barriers

From the width of the respective Lorentzians, the relaxation time (τ), which corresponds to the average time in between reorientational jumps, can be estimated using the following relation

3 $$\tau =\frac{2\hbar }{\mathrm{FWHM}}$$

where ℏ is the reduced Planck constant. The extracted relaxation times are presented in Figure 8 for the NH_3_ ligands as well as for the fast and slow fractions of the BH_4_^–^ anions together with fits of the data to the Arrhenius equation τ = τ_0_e^–*E*_b_/*k*_B_ *T*^, where τ_0_ is a prefactor, *E*_a_ is the energy barrier of the rotational motion, and *k*_B_ is the Boltzmann constant. In addition to the relaxation times for Mg­(BH_4_)_2_·NH_3_, the relaxation time for BH_4_^–^ for α-Mg­(BH_4_)_2_ from ref 58 is presented as a reference. As can be seen, the NH_3_ ligand rotations are significantly faster than those of the fast fraction of the BH_4_^–^ anions. In a similar manner, the slow BH_4_^–^ anions are considerably more sluggish than the fast BH_4_^–^ anions. Comparing the BH_4_^–^ anion dynamics in Mg­(BH_4_)_2_·NH_3_ to α-Mg­(BH_4_)_2_, it can be seen that the relaxation time for the BH_4_^–^ anion in α-Mg­(BH_4_)_2_ (bridging anion) lies between the fast and slow BH_4_^–^ anions in Mg­(BH_4_)_2_·NH_3_. From the fits of the Mg­(BH_4_)_2_·NH_3_ data to the Arrhenius equation, the energy barriers could be determined for the NH_3_ ligand rotation (10 ± 3 meV), the fast BH_4_^–^ anion rotation (63 ± 4 meV), and the slow BH_4_^–^ anion rotation (285 ± 65 meV). In a recent study on Y­(BH_4_)_3_·*x*NH_3_ (*x* = 0, 3 or 7), it was found that the energy barrier of the BH_4_^–^ anion decreases with increasing NH_3_ ligand content and it was suggested that this could be related to the fact that, in Y­(BH_4_)_3_, the BH_4_^–^ acts as a bridging anion between two Y^3+^ cations, while in Y­(BH_4_)_3_·3NH_3_, the BH_4_^–^ acts as a terminal anion coordinating to one Y^3+^ cation and finally in Y­(BH_4_)_3_·7NH_3_, the BH_4_^–^ anion does not directly coordinate to any Y^3+^ cations, but is shielded by the weakly coordinating NH_3_ ligands.^49^ The energy barrier of the slow BH_4_^–^ anions compare relatively well to the energy barrier found for BH_4_^–^ anions in Y­(BH_4_)_3_(∼440 meV), where the anions are bridging two Y^3+^ cations, while the energy barrier of the fast BH_4_^–^ anions is similar to that of the BH_4_^–^ anions in Y­(BH_4_)_3_·3NH_3_ (∼40 meV) where the BH_4_^–^ anions are terminal anions and coordinate to a single Y^3+^ cation.^49^ Based on the observations from ref 49, it can be assumed that the observed fast BH_4_^–^ anions dynamics in Mg­(BH_4_)_2_·NH_3_ corresponds to the terminal BH_4_^–^ anions, while the slow dynamics corresponds to the bridging BH_4_^–^ anions. The clear enhancement of the BH_4_^–^ anion dynamics observed here and in Y­(BH_4_)_3_·*x*NH_3_ and Mg­(BH_4_)_2_·CH_3_NH_2_ ^31,49^ is in sharp contrast to what has been observed for Mg­(BH_4_)_2_·3C_4_H_8_O, where no enhancement of the anion dynamics was observed.^58^ Thus, it can be concluded that while additions of ligands can enhance the dynamics of the BH_4_^–^ anion, this is dependent on how the ligand changes the local environment of the BH_4_^–^ anion. As a comparison to the reorientational energy barriers determined from the QENS data for Mg­(BH_4_)_2_·CH_3_NH_2_, the reorientational energy barriers were estimated from DFT calculations using the cNEB method to be 46.6 meV for the NH_3_ ligand, 161.5 meV for the terminal BH_4_^–^ anion, and 263.4 meV for the bridging BH_4_^–^ anion. The DFT barriers thus exhibit the same trend as the barriers determined from QENS, with a very low barrier for the NH_3_ ligand reorientations followed by two separate energy barriers corresponding to the two different BH_4_^–^ anion local environments. The DFT calculations also suggest that the terminal BH_4_^–^ anion has a lower barrier as compared to the bridging BH_4_^–^ anion. While the DFT calculations are in good agreement with the absolute value determined from QENS for the bridging BH_4_^–^ anion, it estimates a significantly larger barrier for the terminal BH_4_^–^ anion and the NH_3_ ligand as compared to QENS. To explain the discrepancies, we note that the DFT calculations of the energy barriers only consider a quasi-static scenario of each individual entity (NH_3_ or BH_4_^–^), with the neighboring entities only responding to the reorientation of the evaluated entity. In contrast, for a QENS measurement at finite temperatures, the reorientational dynamics of neighboring entities may be coupled, resulting in correlated reorientation, which effectively lowers the energy barrier. Based on the crystal structure, this coupling is likely to happen between the NH_3_ ligands and the terminal BH_4_^–^ anions, but not the bridging BH_4_^–^ anions.

**Figure 8:**
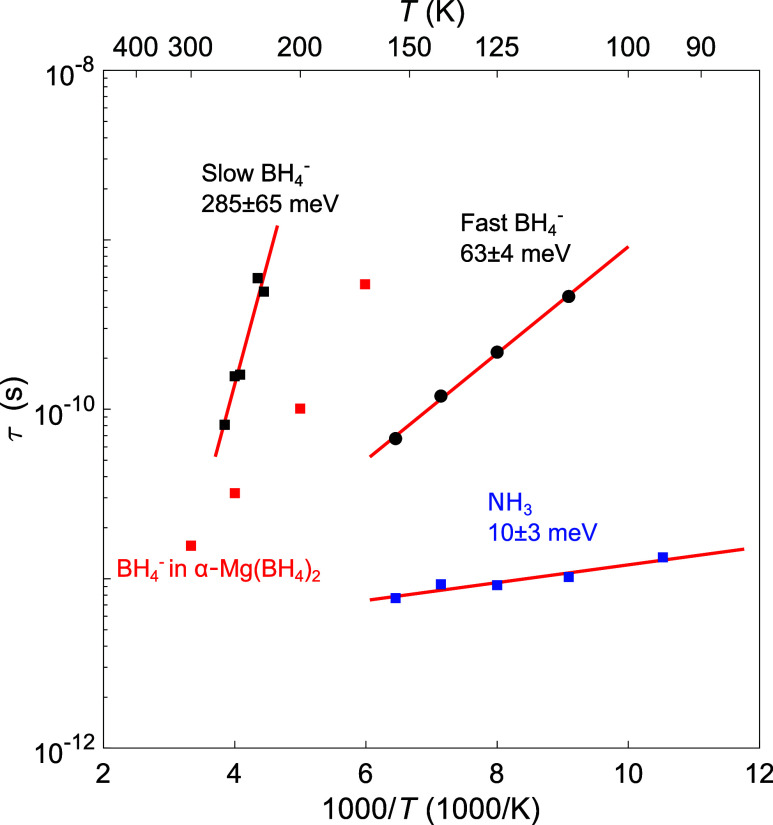
Arrhenius plot of the relaxation time τ for the two populations of dynamically active BH_4_^–^ anions as well as for the NH_3_ ligands. The red lines correspond to fits of the data to the Arrhenius equation. α-Mg­(BH_4_)_2_ data (red squares) adapted from ref 58. Copyright 2019 The Royal Society of Chemistry.

#### 3.3.5 Quantum Mechanical Rotational Tunneling

To further investigate the tunneling peaks observed for the lowest temperatures, QENS spectra measured using DNA (Si111) at 10, 20, 35, and 50 K were fitted using a δ and two Gaussian functions, one for each tunneling peak and a linear background. The δ and Gaussian functions were convoluted with the experimental resolution function. The fits for 10, 20, 35, and 65 K are presented in Figure 9, where the Gaussian components of the fit are highlighted in blue. As can be seen, the peaks start to merge with increasing temperature, and at 65 K, it is no longer possible to tell the peaks apart from normal QENS broadening. In the similar compounds Y­(BH_4_)_3_·3NH_3_ and Y­(BH_4_)_3_·7NH_3_, it was shown using selectively deuterated samples that the tunneling peaks were related to the NH_3_ ligands.^49^ It is therefore assumed that the NH_3_ ligands are also the source of the tunneling peaks in Mg­(BH_4_)_2_·NH_3_. From the detailed QENS spectra at 10 K, it is possible to extract the Q-dependence of the tunneling peaks and estimate the tunneling EISF from

4 $$\mathrm{EIS}F_{\mathrm{tunneling}}=\frac{A_{E}\left(Q\right)}{A_{E}\left(Q\right)+\sum A_{T}\left(Q\right)}$$

where *A*_E_ is the elastic scattering (area of the *delta*-function convoluted with the instrument resolution) and *A*_T_ is the tunneling scattering. For 3-fold rotational tunneling of an NH_3_ ligand around its *C*_3_ axis, the tunneling EISF is as follows

5 $${\mathrm{EISF}}_{t\mathrm{unneling},{\mathrm{NH}}_{3}}=\frac{5+4j_{0}\left(\mathrm{Qd}\right)}{9}$$

**Figure 9:**
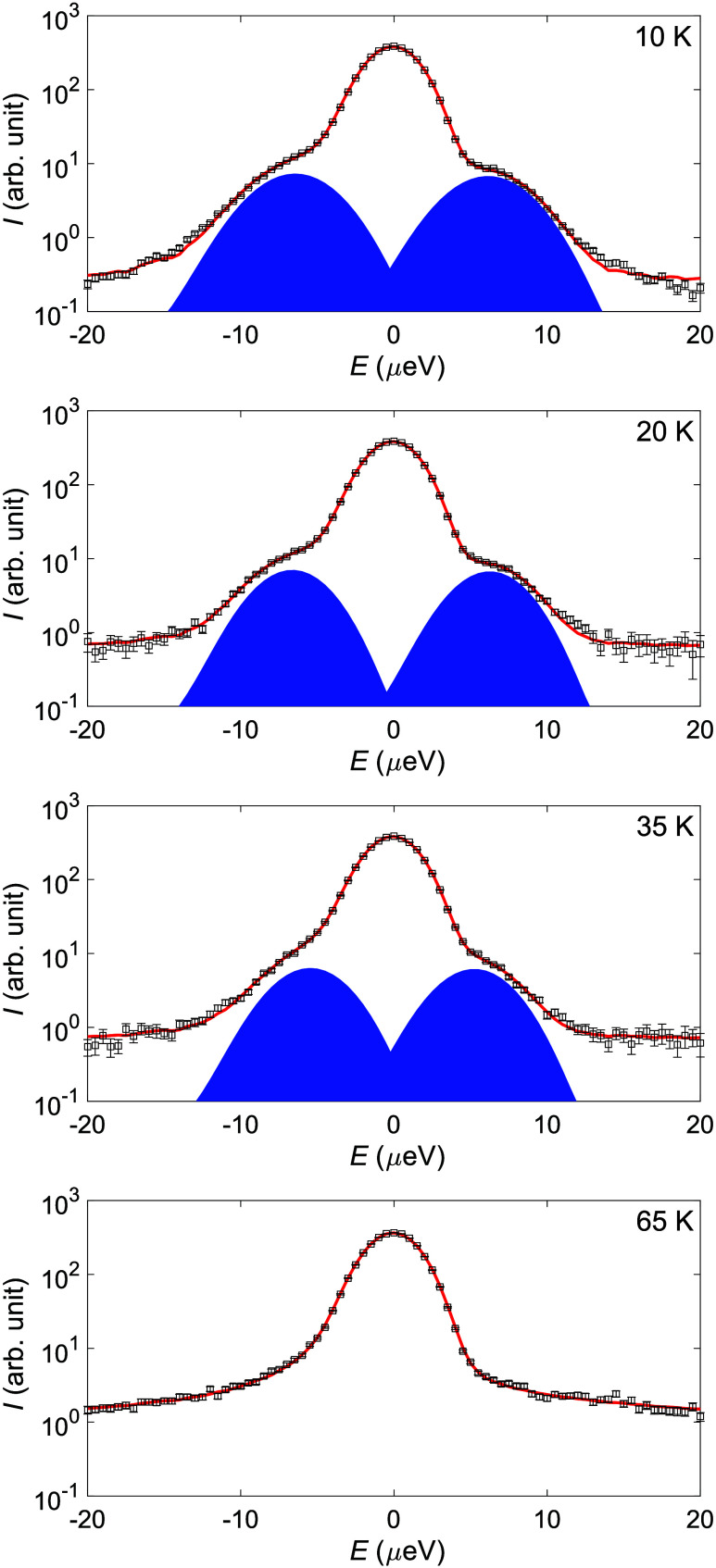
Low-temperature QENS spectra at 10, 20, 35, and 65 K with fits (red line) of the data (black squares). The components corresponding to the quantum mechanical tunneling peaks are highlighted (blue).

where *j*_0_ is the spherical Bessel function of zeroth order and d is the jump distance of the tunneling H atoms in the NH_3_ molecule.^50^ A relatively good agreement is found between the experimental data and the EISF model for 3-fold rotation tunneling of the NH_3_ molecules, as shown in Figure 10. However, a deviation between the data and the EISF model can be observed at low *Q*. This is likely an effect related to multiple scattering of the sample, which is generally more pronounced at lower *Q*.^59^ This effect can also be seen in Figure 7 in the low-*Q* region, further indicating that this is related to multiple scattering. The tunneling EISF suggests that all of the NH_3_ ligands undergo rotational tunneling and that the local environment of the NH_3_ must be very similar, as only one set of tunneling peaks appears and the tunneling energies are very sensitive to the local environment of the tunneling species.

**Figure 10:**
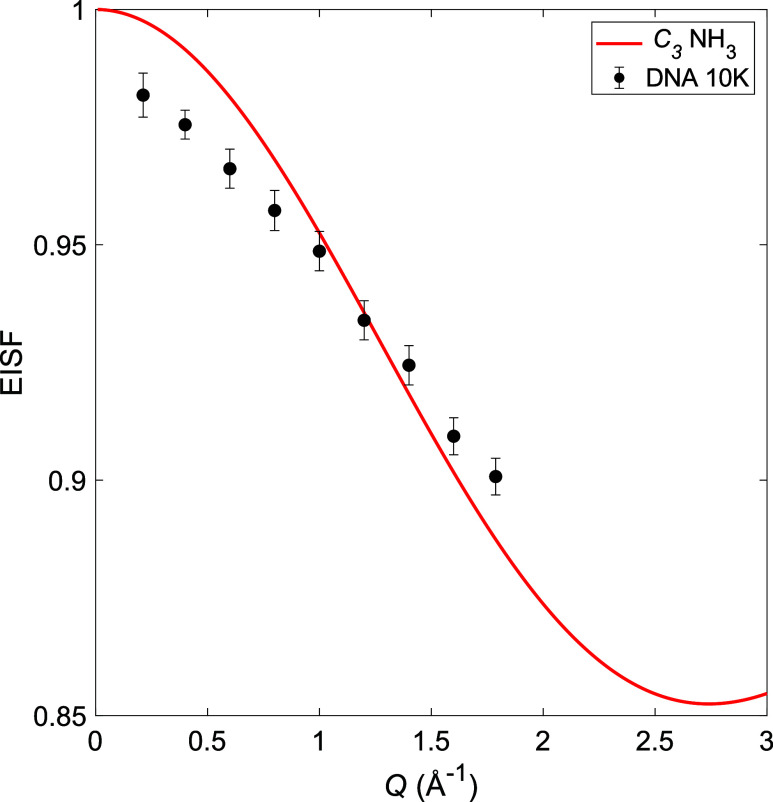
Tunneling EISF for Mg­(BH_4_)_2_·NH_3_ extracted from QENS data at 10 K (DNA Si111).

### 3.4 Ionic Conductivity

The ionic conductivity of Mg­(BH_4_)_2_·NH_3_ was investigated using EIS in the temperature range from 263 to 333 K. From 297 to 333 K, the ionic conductivity is slightly higher than previously reported,^20^ see Figure 11, which may be due to minor differences in sample composition. Comparing the ionic conductivity of Mg­(BH_4_)_2_·NH_3_ to that of pure Mg­(BH_4_)_2_, a significant increase is observed.^20^ The activation energy of Mg­(BH_4_)_2_·NH_3_ was determined to be 1.89 eV (182 kJ mol^–1^) in this temperature range, see Figure S2, and is slightly lower than previously reported, ∼2.3 eV, possibly also owing to small differences in the samples. During heating, a non-Arrhenius behavior is observed in the temperature range of 263–297 K, which suggests a change in the Mg^2+^ cation conduction mechanism during the second-order phase transition observed between 260 and 280 K. Similar ionic conductivity regimes have been observed in other compounds such as Na_2_(B_12_H_12_)_0.5_(B_10_H_10_)_0.5_,^60^ where site disordering of the cation is suggested to be the reason for the more rapid increase in ionic conductivity. The major difference between the low- and high-temperature polymorphs of Mg­(BH_4_)_2_·NH_3_ is the ordering and disordering, respectively, of the bridging [BH_4_]^−^ units. This may suggest that the correlated motion of the cation and anions may be the dominating reason for the faster increase in conductivity with temperature. A phase transition to a high-temperature polymorph is often associated with a rapid increase in ionic conductivity and a lowering of the activation energy as reported for LiBH_4_ ^61^ and several of the metal *nido*- and *closo*-(carba)­borates.^62–64^ However, all of the above-mentioned systems go from essentially dynamically frozen to dynamically active during the transitions to the high-temperature polymorphs. This is not the case in the present study, which exhibits rapid dynamics also in the low-temperature phase and no clear enhancement of the ionic conductivity during the phase transition. This further points to a link between the ionic conductivity and rapid dynamics of the anion in borohydride-based systems.

**Figure 11:**
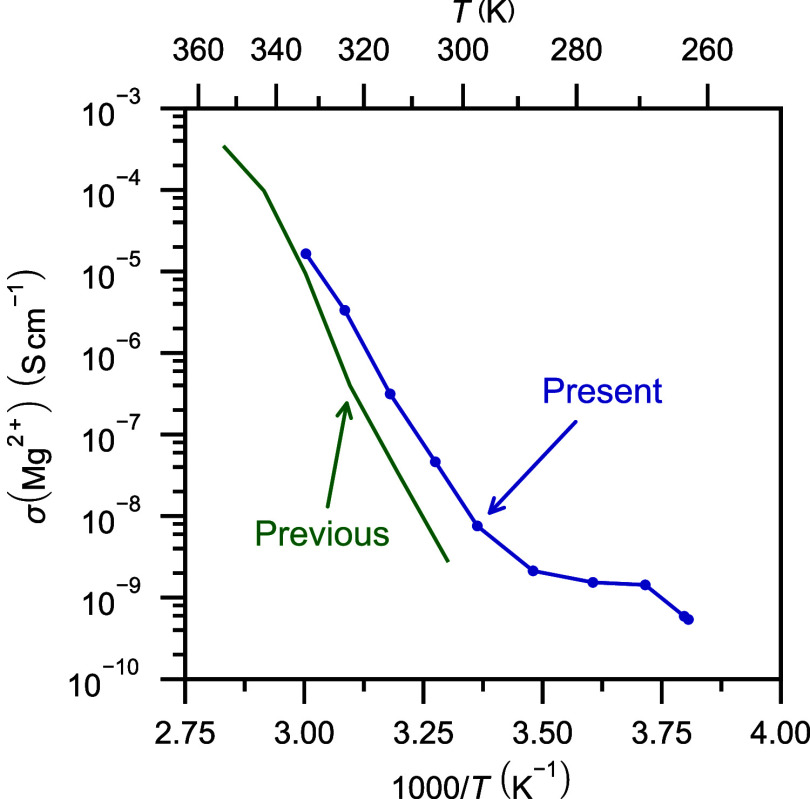
Ionic conductivity of Mg­(BH_4_)_2_·NH_3_. The blue line and data points are from the present study, and the green line represents previously reported data reproduced from ref 20. Copyright 2020 American Chemical Society.

During cooling, the transition observed in the ionic conductivity data differs from that observed during heating. The ionic conductivity drops below the detection limit of the equipment when the sample is cooled to 263 K, see Figure S3. However, when the sample is kept at 263 K, a slow but gradual increase in ionic conductivity is observed until the ionic conductivity reaches the initial ionic conductivity for the heating cycle. It was not possible to obtain a satisfactory fit of the impedance data when the ionic conductivity was below ∼10^–10^ S cm^–1^, but a Nyquist plot of impedance data collected over time at 263 K is provided in Figure S4 to showcase the gradual change at low temperatures.

## 4 Conclusions

The polymorphism, dynamics, and Mg^2+^ ionic conductivity of Mg­(BH_4_)_2_·NH_3_ were investigated using INS, QENS, SR-PXD, EIS, and DFT. The SR-PXD and INS results revealed a new low-temperature polymorph for Mg­(BH_4_)_2_·NH_3_, which exists below ∼270 K and contains two different local environments for the BH_4_^–^ anion. In the first environment, the BH_4_^–^ anion coordinates to a single Mg^2+^ cation (terminal anion), while in the second, it coordinates to two separate Mg^2+^ cations (bridging). The EIS and QENS results showed that incorporation of the neutral NH_3_ ligands enhances both the Mg^2+^ ionic conductivity as well as the reorientational mobility of the BH_4_^–^ anions as compared to Mg­(BH_4_)_2_. The results also show that the two local environments for the BH_4_^–^ anions lead to a significant difference in dynamics, where the terminal BH_4_^–^ anions undergo fast 3-fold rotations around their *C*_3_ axes, while the bridging BH_4_^–^ anions perform slower 2-fold rotations around their *C*_2_ axes. Furthermore, the QENS results show that the NH_3_ ligands are also dynamically active and undergo very fast 3-fold rotations around their *C*_3_ axes. Below 50 K, all of the NH_3_ ligands exhibit quantum mechanical rotational tunneling with a single well-defined tunneling peak pair, which implies that the local environment is close to identical for all of the NH_3_ ligands.

The increase in the reorientational dynamics of the BH_4_^–^ anion concomitant with a large increase in the Mg^2+^ ionic conductivity in both the low- and high-temperature polymorphs, similar to the behavior observed for Mg­(BH_4_)_2_·CH_3_NH_2_,^31^ implies a link between the reorientational dynamics and the high ionic conductivities in this material class. Furthermore, the increase in reorientational dynamics is only seen for the terminal BH_4_^–^ anions, suggesting that the dynamics of these anions may be the primary contributors to the enhancement of the ionic conductivity.

## Supplementary Material



## Notes

SR-PXD data, EIS data, as well as mathematical expressions of relevant EISF models (PDF)
