# Supplementary material for: Concomitant Enhancement of the Reorientational Dynamics of the BH4 – Anions and Mg2+ Ionic Conductivity in Mg(BH4)2·NH3 upon Ligand Incorporation
Source: J Phys Chem C Nanomater Interfaces. 2025 Dec 23;130(1):112–23. doi: 10.1021/acs.jpcc.5c07031 (PMC12797288; doi:10.1021/acs.jpcc.5c07031)
Supplement: Supplementary file 1 [file jp5c07031_si_001.pdf]

Supporting Infomation
Concomitant enhancement of the reorientational dynamics of the BH_4^- anions and Mg^{2+} ionic conductivity in $Mg(BH_4)_2{\cdot}NH_3$ upon ligand incorporation

J. B. Grinderslev,[1] M. B. Amdisen,[1] S. Rosenqvist Larsen,[2]
B. A. Trump,[3] M. Karlsson,[4] W. Zhou,[3] T. J. Udovic,[3]
Y. Cheng,[5] T. Tominaga,[6] T. R. Jensen,[1] and M. S. Andersson[2,*]

[1]*Interdisciplinary Nanoscience Center (iNANO) and Department of Chemistry, University of Århus, Langelandsgade 140, DK-8000 Århus C, Denmark*
[2]*Department of Chemistry - Ångström Laboratory, Uppsala University, Box 538, SE-751 21 Uppsala, Sweden*
[3]*NIST Center for Neutron Research, National Institute of Standards and Technology, Gaithersburg, Maryland 20899-6102, United States*
[4]*Department of Chemistry and Chemical Engineering, Chalmers University of Technology, SE-412 96 Göteborg, Sweden*
[5]*Neutron Scattering Division, Oak Ridge National Laboratory, Oak Ridge, Tennessee 37831, United States*
[6]*Research Center for Neutron Science and Technology, Comprehensive Research Organization for Science and Society (CROSS), Tokai, Ibaraki 319-1106, Japan*

I. SYNCHROTRON X-RAY POWDER DIFFRACTION

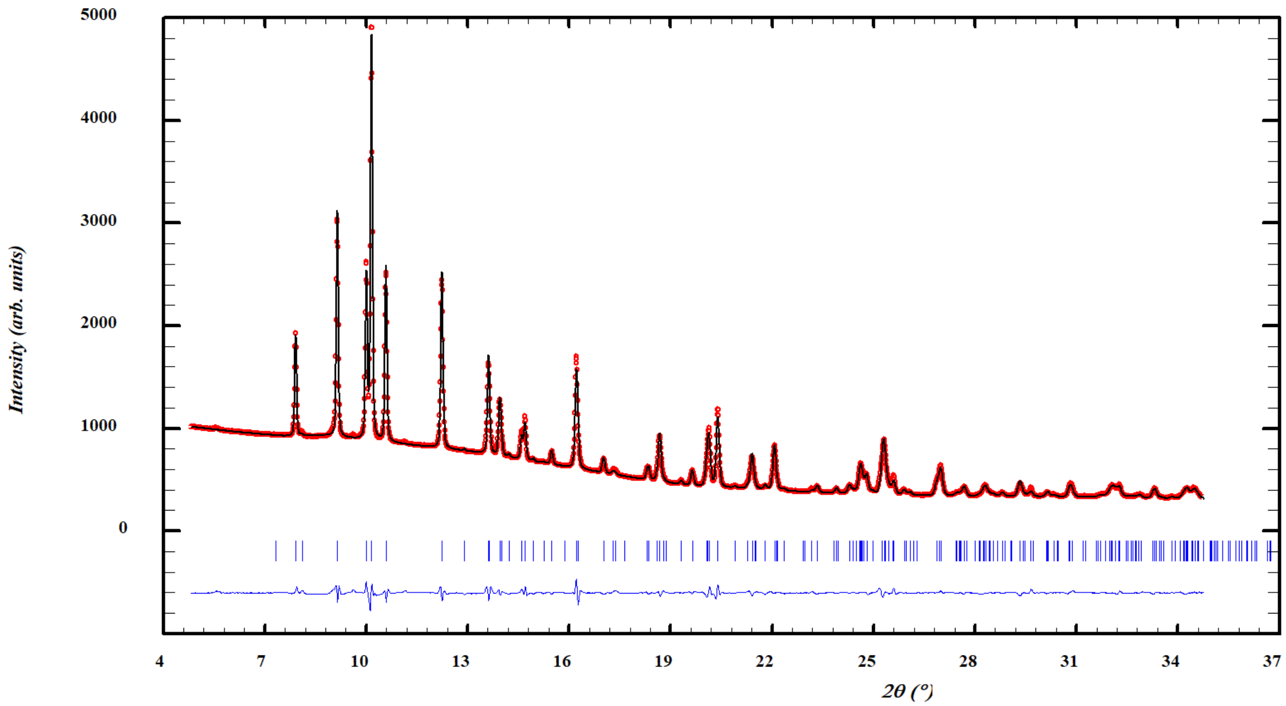

Figure S1. Rietveld refinement of the structural model for the low-temperature polymorph of $Mg(BH_4)_2 \cdot NH_3$ ($P2_12_12_1$) from SR-PXD data measured at 150 K, $\lambda = 0.7980$ Å, showing experimental (red circles) and calculated (black line) PXD patterns, and a difference plot below (blue line). Blue tick marks: $Mg(BH_4)_2 \cdot NH_3$. Final discrepancy factors: $R_p = 1.21$ %, $R_{wp} = 1.90$.

* mikael.andersson@kemi.uu.se

II. IONIC CONDUCTIVITY

The activation energy of $Mg(BH_4)_2 \cdot NH_3$ was determined from a $\ln(\sigma T)$ vs. $1000/T$ plot (Fig. S2) and the equation

$$\sigma = \frac{\sigma_0}{T} e^{\frac{-E_\mathrm{a}}{k_\mathrm{B} T}} \tag{1}$$

where σ is the ionic conductivity, σ_0 is a prefactor, T is the temperature, E_a is the activation energy, and k_B is the Boltzmann constant.

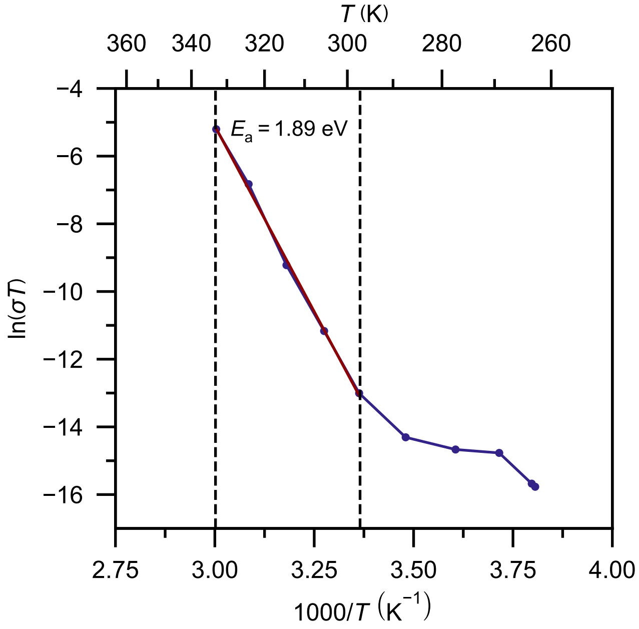

Figure S2. $\ln(\sigma T)$ vs. $1000/T$ plot of $Mg(BH_4)_2 \cdot NH_3$ ionic conductivity data for activation energy determination. The red line shows the linear fit within the temperature range marked by the dotted vertical lines.

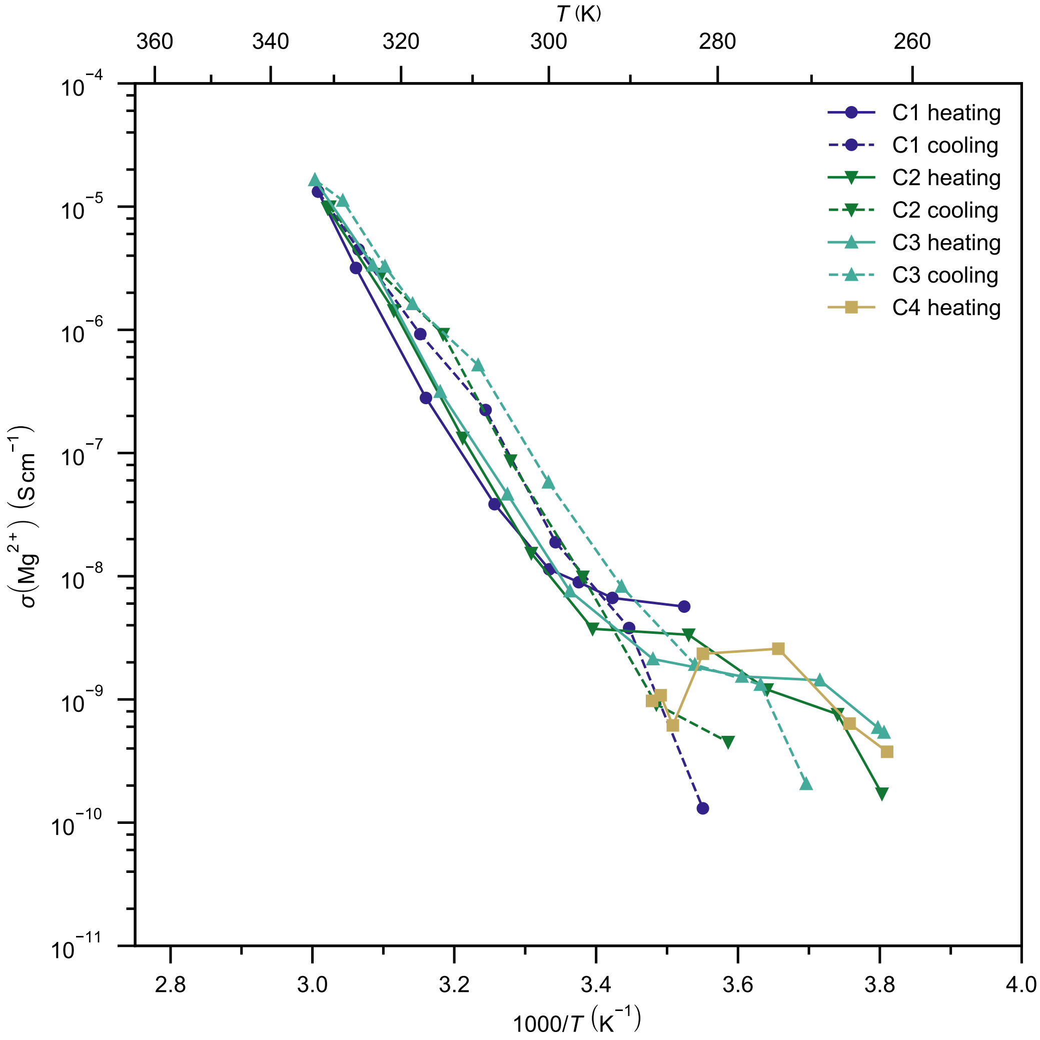

Figure S3. Temperature-dependent ionic conductivity of $Mg(BH_4)_2 \cdot NH_3$ over multiple heating-cooling cycles (C).

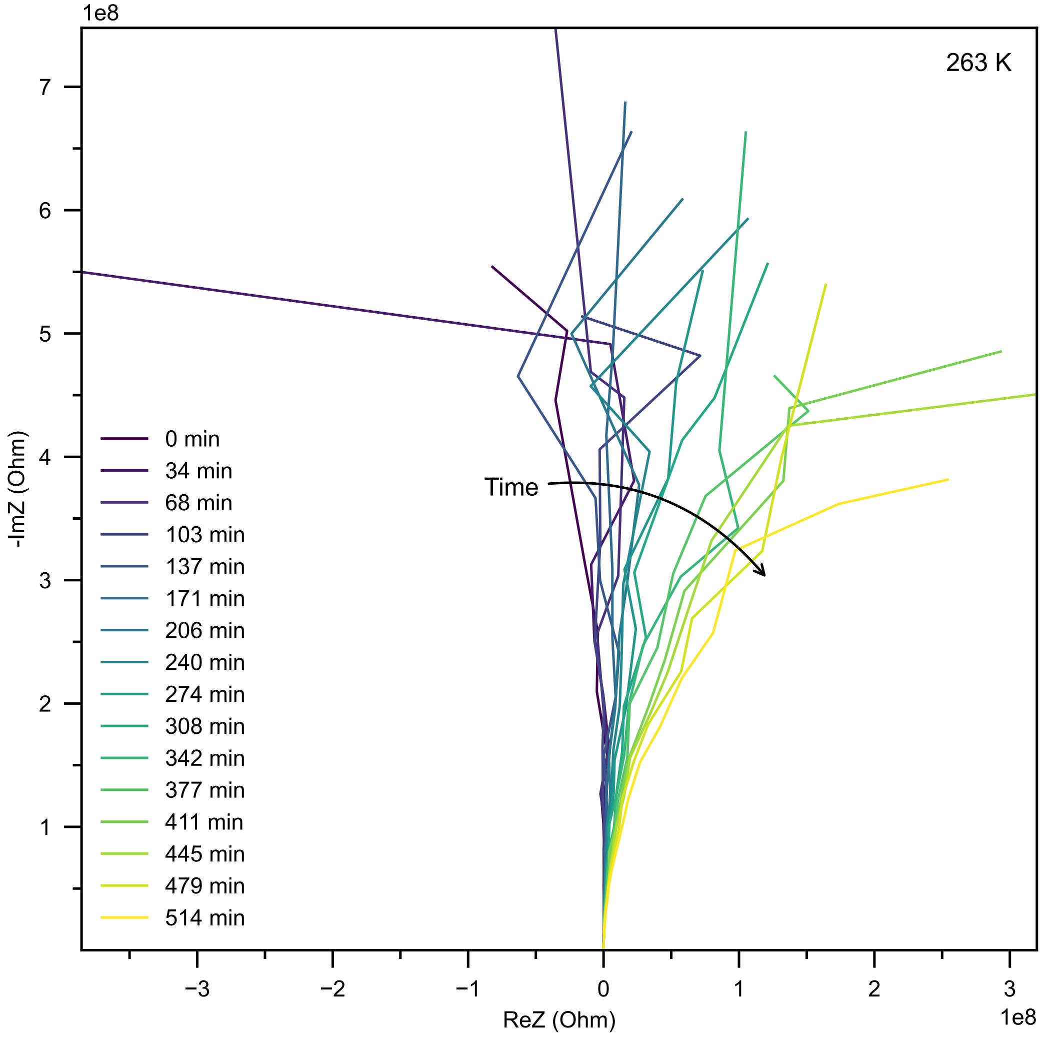

Figure S4. Nyquist plot of EIS data over time for $Mg(BH_4)_2 \cdot NH_3$ at 263 K.

III. EISF MODEL CURVES FOR NH_3 AND BH_4^-

The EISF models C_2 or C_3 reorientation,[S1] tetrahedral tumbling [S2] and cubic tumbling[S3] for a tetrahedral molecule or ion such as BH_4^- are given by:

$$\mathrm{EISF}_{C_2/C_3,\ \mathrm{BH}_4^-} = \frac{1 + j_0(Qd)}{2}, \tag{2}$$

$$\mathrm{EISF}_{\mathrm{tetrahedral},\ \mathrm{BH}_4^-} = \frac{1 + 3j_0(Qd)}{4}, \tag{3}$$

$$\mathrm{EISF}_{\mathrm{cubic},\ \mathrm{BH}_4^-} = \frac{1 + 3j_0(Qd/\sqrt{2}) + 3j_0(Qd) + j_0(Qd\sqrt{3/2})}{8}, \tag{4}$$

where d is the jump distance and $j_0 = \sin(x)/x$ is the zeroth-order spherical Bessel function. For a trigonal pyramidal molecule, such as NH_3 the EISF model for C_3 reorientation[S4] is given by:

$$\mathrm{EISF}_{C_3,\ \mathrm{NH}_3} = \frac{1 + 2j_0(Qd)}{3}, \tag{5}$$

In the case of NH_3 and BH_4^-, d is the same as the H-H distance in the respective molecule/ion (1.64 Å in NH_3, 1.96 Å in BH_4^-). Due to the large incoherent neutron scattering cross section of H in comparison to the other elements (Mg, ^{11}B and N) only hydrogen dynamics need to be taken into account when calculating the EISF.

References

S1. Yildirim, T.; Gehring, P. M.; Neumann, D. A.; Eaton, P. E.; Emrick, T. Neutron-scattering investigation of molecular reorientations in solid cubane. *Phys. Rev. B* **1999**, *60*, 314–321
S2. Sköld, K. Effects of molecular reorientation in solid methane on the quasielastic scattering of thermal neutrons. *J. Chem. Phys.* **1968**, *49*, 2443–2445
S3. Rush, J. J.; de Graaf, L. A.; Livingston, R. C. Neutron scattering investigation of the rotational dynamics and phase transitions in sodium and cesium hydrosulfides. *J. Chem. Phys.* **1973**, *58*, 3439–3448.
S4. Bée, M. *Quasielastic Neutron Scattering*; Adam Hilger, Bristol, 1988
